# Risk for molecular contamination of tissue samples evaluated for targeted anti-cancer therapy

**DOI:** 10.1371/journal.pone.0173760

**Published:** 2017-03-13

**Authors:** Eyal Asor, Michael Y. Stav, Einav Simon, Ibrahim Fahoum, Edmond Sabo, Ofer Ben-Izhak, Dov Hershkovitz

**Affiliations:** 1 B. Rappaport Faculty of Medicine, Technion-Israel Institute of Technology, Haifa, Israel; 2 Institute of Pathology, Rambam Health Care Campus, Haifa, Israel; 3 Institute of Pathology, Tel-Aviv Sourasky Medical Center, Tel-Aviv, Israel; 4 Sackler Faculty of Medicine, Tel-Aviv University, Tel-Aviv, Israel; Tel Aviv University, ISRAEL

## Abstract

With the increasing usage of sensitive PCR technology for pharmacogenetics, cross contamination becomes a significant concern. Researchers employed techniques which basically include replacing laboratory equipment after each sample preparation; however, there are no recommended guidelines. In the present work we wanted to evaluate the risk of cross contamination during tissue processing using the routine precaution measures. Twenty-one surgical samples of lung adenocarcinoma were used, of which 7 contained *EGFR* exon 19 mutation, 7 contained *EGFR* exon 21 mutation (p.L858R) and 7 were *EGFR* wild-type. The samples were ordered by alternating the mutation group to maximize the potential for cross contamination and underwent tissue sectioning and de-paraffinization. The entire process was performed using the same tools. Following DNA extraction all samples underwent PCR amplification and were scrutinized for small fractions of *EGFR* mutation using deep sequencing with the Ion torrent PGM technology. Twenty samples yielded results. The fraction of mutated copies was 41 ± 23% (range 11–66) for the cases with known exon 19 mutation and 48±24% (range 0–65) for the cases with known exon 21 mutations. No in-frame exon 19 deletion mutations were identified in the wild-type (WT) and exon 21 groups. The fraction of *EGFR* exon 21 (codon 858) mutations was 0.018±0.014% (range 0–0.05%) in the WT and exon 19 groups, which was not statistically different than the background sequencing artifact noise for the same base-pair alteration (p = 0.21). Our results suggest that standard precautions are sufficient for molecular pathology diagnosis of surgical samples and are not associated with increased risk of cross contamination.

## Introduction

During the last decade, the availability and application of molecular pathology techniques in medical institutions has increased considerably [1]. Advances in mutation analysis methods made it possible to establish diagnosis, predict disease course and determine treatment response based on analysis of small number of tumor cells [2]. Examples for treatment directed by mutation analysis include the use of Tyrosine Kinase Inhibitors (TKI) for the treatment of non-small cell lung carcinoma (NSCLC) carrying specific Epidermal growth factor receptor (*EGFR*) gene mutations [3], or treatment of colorectal cancer with anti-EGFR therapy in cases without *KRAS* gene mutations [4]. Diagnosis of these mutations is made prior to treatment decision using sensitive molecular methods including Polymerase Chain Reaction (PCR) and next-generation sequencing (NGS) [5]. The volume of diagnostic molecular pathology testing is rising rapidly and molecular pathology laboratories are expected to provide reliable and high quality diagnoses [6].

A major pitfall in general pathology is cross contamination [7], this problem is even more pronounced in molecular pathology since the basic methodology involves considerable amplification of small nucleic acid fragments. Cross contamination can take place in any stage of tissue processing; gross dissection in the operating room, tissue processing for microscopic evaluation in the laboratory and during DNA extraction procedure which may lead to wrong template amplification and false positive or negative results. Remains of extraneous tissue, known as “floaters” are small tissue fragments being carried over from one case to the next during processing in the laboratory. In order to overcome the problem of cross contamination by floaters, some methods have been applied such as immunohistochemical staining of paraffin embedded tissue samples with ABH blood group antibodies [8], microdissection and microsatellite analysis [9]. However, laboratory errors related to floater cross contamination are not uncommon [10]. Gephardt and Zarbo reported that the frequency of floaters contamination from two hundred seventy-five surgical pathology laboratories institutions from North America is up to 2.9% [11]. Layfield and his colleagues also studied floaters contamination in revision of more than half a million slides, and reported that the frequency decreased in prospectively inspected slides [12]. Nevertheless, in molecular pathology the risk for contamination might be higher due to the amplification process. Another example of cross contamination comes from cell line research studies. KU7 (a clone of bladder cancer cell line) was found to be contaminated with HeLa (a clone of cervical cancer cell line) and consequently damaged the value of 30 years study results using this cell line throughout the world [13]. Cross contamination pitfalls are not limited to the field of cancer alone. Varanat and his colleagues found molecular remains of Bartonella DNA dissemination and transfer in the necropsy room and during the subsequent processing of Formalin Fixed Paraffin Embedded (FFPE) tissues in veterinary pathology [14]. Ritchey and his colleagues reported that viral DNA can contaminate a microtome knife so that subsequently sectioned un-inoculated control tissues exhibit false positive PCR amplification [14].

In order to avoid the cross contaminations, certain guidelines have been proposed for DNA extraction kits and PCR protocols [15,16]. Yet, regarding the process of routine FFPE sample preparation, such defined recommendations are absent. Tissue processing for histological studies usually includes—fixation, trimming, dehydration, clearing, infiltration, embedding, de-paraffinization and staining. These steps have been used for over a century without any substantial changes. Routinely, different tissues, from different patients processed in the same working area (the basins used for fixation, dehydration etc.). In addition, the same knife blade is used for cutting different tissue samples. Since laboratory molecular cross contamination is a major concern, safety measures are taken in order to reduce this risk. These measures vary between the laboratories. Several labs replace the microtome blade with each new sample section [17–19], while others add an step of extensive microtome washing with DNA decontamination solution [17] or a “sandwich cutting technique”, which means that an empty paraffin blocks were cut in between patient tissue samples [20]. Other works do not provide detailed information about their cross contamination prevention technique [21,22].

Molecular pathology misdiagnoses might have significant impact on patients' treatment and survival and therefore every effort should be made to prevent cross contamination. However cross contamination prevention guidelines should be based on consistent data. Moreover, the standardized guidelines should be suitable for the growing workload of the molecular pathology laboratories. The aim of our present study was to examine a basic question, does the conventional tissue processing method carry a risk for molecular cross contamination.

## Materials and methods

This study was approved by the local ethics committee.

### Tissue samples

Twenty one cases of lung adenocarcinoma samples were included, all taken between the years 2007–2011. Of these 21 samples, 7 carried *EGFR* exon 19 deletion mutation and 7 carried *EGFR* exon 21 point mutation (p.L858R), which account for about 90% of the observed *EGFR* mutations in adenocarcinoma of the lung [3]. The other samples were *EGFR* wild-type (WT).

### Tissue preparation

The samples underwent the conventional histopathological tissue preparation. Briefly, slides were cut using a microtome and following de-paraffinization, areas containing more than 50% tumor cell fraction were scraped and underwent DNA extraction.

### Study procedure

**Fig 1:**
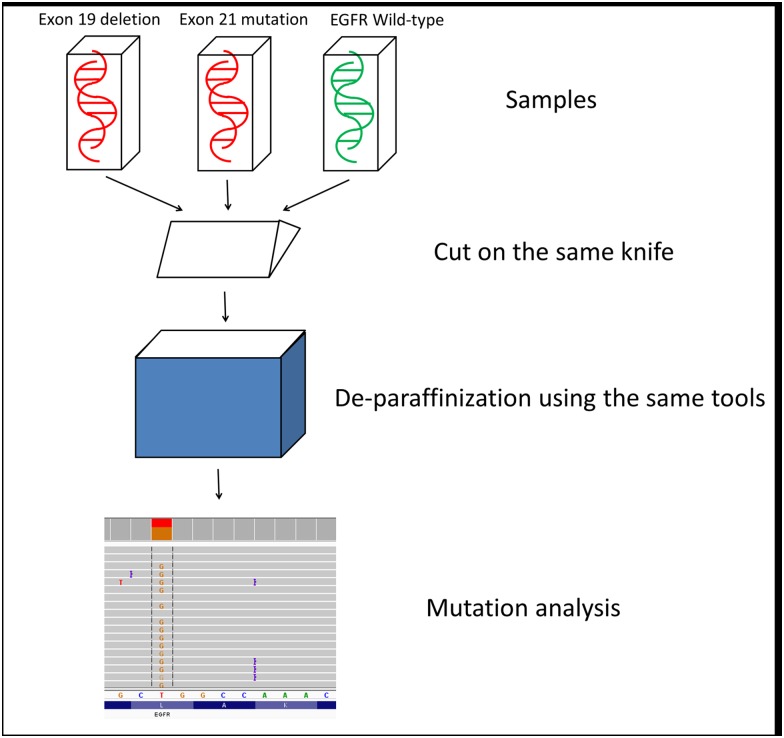
Study workflow. Study procedure was divided to three main stages: 1) Sequential cutting of the samples using the same knife 2) De-paraffinization using the same tools 3) DNA extraction and mutation analysis.

To evaluate the possibility of cross contamination during the pre-analytical process, all samples were sequentially cut on the same knife and underwent de-paraffinization in the same tools. Additionally, to maximize the potential for cross contamination, samples were ordered in by alternating mutation group (exon 19 mutation followed by exon 21 mutation followed by WT and so forth) (Fig 1).

### Determining detection limit of the test

Classically, the limit of detection for point mutations using the Ion-Torrent PGM is 1% [23]. Using ultra-deep sequencing it should be possible to identify even lower frequency alleles, however this might be limited by the level of sequencing and PCR artifacts [24]. To determine the "noise" level of our method we calculated the distribution of non-reference change in the bases sequenced (excluding "hot-spot" positions). A signal at the "hot-spot" position higher than the 95^th^ percentile background "noise" level will be defined as the detection limit of the method (a value higher than 95^th^ percentile in the "hot-spot" position has a chance of less than 5% to be an artifact—p<0.05).

### Cross contamination assessment

For the identification of the small fractions of possible contamination mutant DNA, we used the Ion Torrent Personal Genome Machine (PGM) sequencer platform. DNA extracted from the tissue and 3-25ng of DNA (measured using Qubit 2.0 Fluorometer, Life Technologies, Carlsbad, CA) was used per reaction (corresponding to DNA content from 500–4107 cells).

PCR amplification was performed using the primers for the selected mutation (Table 1). Analysis of DNA quality using TapeStation (Agilent, Santa Clara, CA) showed that DNA fragment length in the samples was 467±185 (range 260-997bp), indicating that more than 50% of DNA fragments in our samples could be amplified in our PCR reaction (amplicon length 224bp and 208bp for exons 19 and 21, respectively).

**Table 1:** Primers list.

EGFR mutation	Forward	Reverse
exon 19	AGCATGTGGCACCATCTCAC	AGACATGAGAAAAGGTGGGC
exon 21	AATTCGGATGCAGAGCTTC	GCATGGTATTCTTTCTCTTCCG

Each primer pair was supplemented with Ion-Torrent adapters P1 and A, to allow binding to the Ion Sphere Particles (ISPs). Additionally, 21 different forward primers, each with a different barcode, were used for every case to allow the analysis of multiple samples in a single reaction. Amplicons were purified using the Qiagen PCR purification kit (Qiagen, Hilden, Germany) and were deep-sequenced using an Ion 314 chip on the PGM for 65 cycles. Data from the PGM runs was first processed using the Ion Torrent platform-specific pipeline software Torrent Suite v1.3.1 to generate sequence reads, trim adapter sequences, filter, and remove poor signal-profile reads. The algorithm used is suitable for identifying somatic substitutions and deletions on the Ion-Torrent PGM. Generated sequence files were aligned to the genomic sequence of *EGFR* exons 19 and 21 and we determined the presence of low fraction mutations in each sample using the Integrative Genomic Viewer (IGV 2.3) free software [25,26].

### Statistical analysis

Power analysis: In order to identify errors in diagnosis in 5% of cases with a statistical significance (α type of error) of 0.05 and a statistical power of 90% (β type of error of 0.1), we need a sample size of 32 cases. In this study, we made an analysis that included 42 reactions (21 patients and 2 mutations per patient), hence the sample size was satisfying. Differences between percentages of molecular contamination in different genetics sites were analyzed by Wilcoxon test. P. values < 0.05 were considered as statistically significant.

## Results

Overall 21 DNA samples underwent sectioning, de-paraffinization and DNA extraction and PCR. One sample from the *EGFR* exon 21 mutation group failed PCR amplification and was excluded from the study. This is likely the results of poor DNA quality, either due to in-appropriate tissue fixation or due to some technical problem during DNA extraction. Additionally, sample 12, which was expected to harbor mutation c.2573T>G based on previous mutation analysis of a different tissue block, was found to be wild-type for this mutation. This could represent a case of intra-tumor heterogeneity. DNA concentrations, tumor cell fraction, mutant allele frequency and mutation type are presented in Table 2.

**Table 2:** Mutation characteristics of the samples.

Case	Concentration (ng/ul)	Mutation	Tumor cell fraction	Mutant allele frequency
**1**	54	c.2236_2250del	70	45
**2**	6	WT	30	0
**3**	16	c.2573T>G	20	60
**4**	22	c.2235_2249del	20	17
**5**	30	WT	20	0
**6**	10	c.2573T>G	80	42
**7**	23	c.2236_2250del	80	33
**8**	10	WT	30	0
**9**	N.D.	N/A	30	N/A
**10**	3	c.2235_2249del	40	17
**11**	36	WT	10	0
**12**	6	WT	20	0
**13**	3	c.2235_2249del	70	27
**14**	3	WT	20	0
**15**	6	c.2573T>G	40	64
**16**	9	c.2236_2250del	50	54
**17**	15	WT	60	0
**18**	1	c.2573T>G	30	60
**19**	25	c.2236_2250del	50	43
**20**	11	WT	20	0
**21**	49	c.2573T>G	40	52

Of note, assuming 50% mutant allele frequency in the tumor cells, some samples showed higher than expected mutant allele frequency. For example, while only 20% of the cells in sample 3 were tumor cells the mutant allele frequency was 60%. This is probably the consequence of mutant allele specific amplification, a phenomenon that has been previously described regarding *EGFR* mutations in lung cancer [27,28].

**Fig 2:**
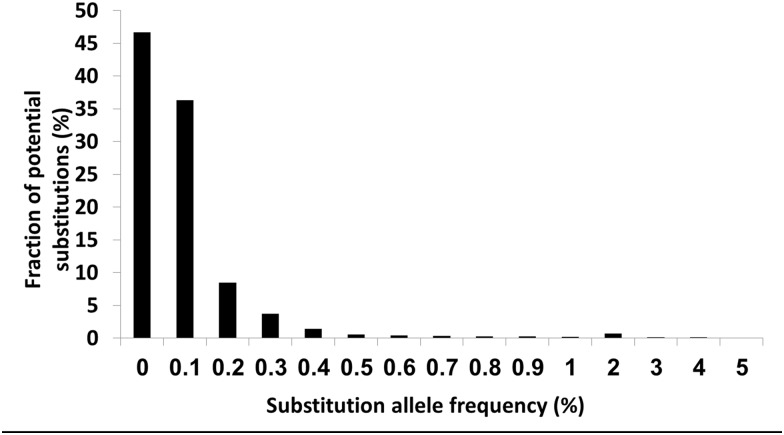
Distribution of the different substitution allele frequencies in the non-”hot-spot” positions. As shown, allele frequencies higher than 0.3% were present in less than 5% of possible positions and we therefore set 0.3% allele frequency as the detection limit of our method.

Analysis of the distribution of background "noise" in the non-"hot-spot" bases showed that sequence alterations with allele frequency of 0.3% or higher were present in less than 5% of the positions (the 95^th^ percentile of substitutions in mom-hot-spot positions was 0.29%), corresponding to p value <0.05. Based on this mutant allele frequency >0.3% in the "hot-spot" position was set as the detection limit of our test (Fig 2).

**Fig 3:**
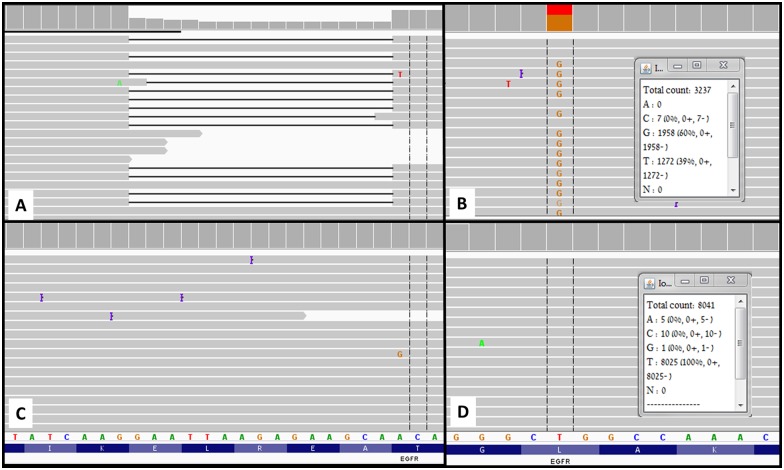
Mutation analysis using the Ion Torrent PGM. (A) A sample with a known *EGFR* exon 19 deletion mutation. (B) A sample with *EGFR* exon 21 mutation (p.L858R) is present (39% T > G transversion). (C) Sequencing result of *EGFR* exon 19 wild-type case with lack of in frame deletion. (D) A sample with wild-type *EGFR* exon 21 "hot-spot" position (100% T, no T >G transversion). Using the Integrative Genomics Viewer (IGV) software, we were able to determine the mutation and fraction of mutant copies.

We determined the presence of low fraction mutations in each sample by using the Integrative Genomics Viewer. The read depth was 4760 ±1481 (range 1779–7059) for *EGFR* exon 19 and 5878.2 ±2838 (range 974–10645) for exon 21. The fraction of mutated copies was 41 ± 23% (range 11–66) for the cases with known exon 19 mutation (Fig 3A) and 48±24% (range 0–65) for the cases with known exon 21 mutation (Fig 3B). No in-frame exon 19 deletion mutations were identified in the WT or exon 21 groups (Fig 3C). The fraction of EGFR exon 21 (p.L858R) mutations was 0.018±0.014% (range 0–0.05%) in the WT and exon 19 groups (Fig 3D).

**Fig 4:**
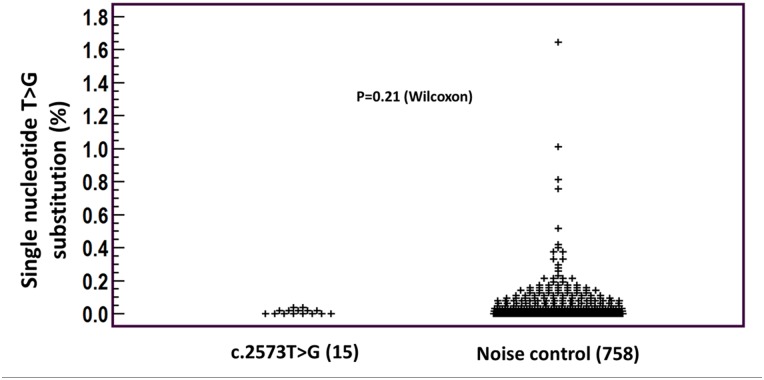
Comparison of EGFR exon 21 "hotspot" locus (c.2573T>G) mutation fraction and sequencing noise. There was no significant difference in the fraction of *EGFR* exon 21 (cDNA position 2573) "hotspot" mutation (T>G) from the same base alteration fraction in the other T residues in the sequence (noise). Statistical significance was assessed by Wilcoxon test.

To evaluate whether our samples contained low frequency contamination we first wanted to determine the level of noise caused by artifacts of the sequencing procedure. Toward this aim we measured the fraction of T>G transversion in all the non-"hot-spot" T residues in our sequences. These positions do not carry driver mutations in cancer and we therefore defined T>G fraction in these positions as the level of sequencing artifacts. The fraction of T>G change was 0.03± 0.09% (range 0–1.64%). No significant difference was found between the fraction of T>G transversion in the "hot-spot" position and the non-"hot-spot" positions (p = 0.21), indicating that the low fraction c.2573T>G in the exon 19 mutation and WT groups are sequencing artifact rather than true low fraction mutation (Fig 4).

## Discussion

The main finding of the present study is that standard precautions in FFPE sample preparation, such as cleaning microtome knife with a brush between samples, replacing it once a day and replacing the fluids in the de-paraffinization basin daily, were not associated with molecular cross contamination.

The accumulating genetic data on different type of diseases together with the progresses in diagnostic tools and therapeutic options intensify the workload burden on the molecular pathology laboratories. To keep pace with this ongoing development, the laboratories need to improve their diagnostic capacity for the increase number of daily samples, yet, without impairment in the current quality. For this purpose, quality control guidelines should be standardized by evidence based data only. Cross contamination is a major concern in molecular pathology and many studies demonstrated how cross contamination does occur in cancer pathological research [8,10,13] as well as microbiology [14,29].

Several labs reported different approaches for cross contamination prevention including frequent microtome blade replacement, the use of empty paraffin blocks between sample block (“sandwich cutting technique”) and the use of DNA decontamination solutions [17–20]. However, each of these techniques is time consuming and reduces the laboratory workload capacity significantly. Over the last decade the increase in pathology workload burden has resulted in a 60% increase slide numbers, 100% increase in immunohistochemistry procedures, and more than 200% increase in molecular analyses, moreover, this workload increment was not accompanied by an increase in laboratory stuff [30]. Hence, additional time consuming and potentially unnecessary steps should be carefully considered before being included in the tissue preparation procedure.

Of note, our study was restricted to the analysis of surgical specimens. These samples are relatively large, and hence might be less sensitive to contamination by minute amount of DNA. On the other hand, larger samples are more likely to cause contamination due to their high cellular content. Nevertheless, further studies are needed to determine whether standard precautions are enough to prevent contamination in small needle biopsies as well. Additionally, the detection limit of our method was 0.3% and it is possible that a contamination resulting in mutant allele frequency lower than 0.3% might go unnoticed by our approach. However, contamination resulting in such a low allele frequency would be irrelevant clinically as the detection limit of most available tools is in the rage of 1–5% [31].

To the best of our knowledge, this is the first research to test the degree of cross contamination in the FFPE processing for molecular diagnosis. Our findings suggest that standard precautions are not associated with increased risk for contamination of surgical pathology specimens. Evaluation of large numbers of surgical specimens for molecular pathology can be safely performed without the need for additional time consuming steps.
